# Bicarbonate boosts flash response amplitude to augment absolute sensitivity and extend dynamic range in murine retinal rods

**DOI:** 10.3389/fnmol.2023.1125006

**Published:** 2023-04-14

**Authors:** Rajan D. Adhikari, Amanda M. Kossoff, M. Carter Cornwall, Clint L. Makino

**Affiliations:** Department of Physiology and Biophysics, Boston University Chobanian and Avedisian School of Medicine, Boston, MA, United States

**Keywords:** cGMP, ERG, cpfl3, Gnat2, membrane guanylate cyclase, visual transduction, neuromodulation, mutant mouse

## Abstract

Rod photoreceptors in the retina adjust their responsiveness and sensitivity so that they can continue to provide meaningful information over a wide range of light intensities. By stimulating membrane guanylate cyclases in the outer segment to synthesize cGMP at a faster rate in a Ca^2+^-dependent fashion, bicarbonate increases the circulating “dark” current and accelerates flash response kinetics in amphibian rods. Compared to amphibian rods, mammalian rods are smaller in size, operate at a higher temperature, and express visual cascade proteins with somewhat different biochemical properties. Here, we evaluated the role of bicarbonate in rods of *cpfl3* mice. These mice are deficient in their expression of functional cone transducin, Gnat2, making cones very insensitive to light, so the rod response to light could be observed in isolation in electroretinogram recordings. Bicarbonate increased the dark current and absolute sensitivity and quickened flash response recovery in mouse rods to a greater extent than in amphibian rods. In addition, bicarbonate enabled mouse rods to respond over a range that extended to dimmer flashes. Larger flash responses may have resulted in part from a bicarbonate-induced elevation in intracellular pH. However, high pH alone had little effect on flash response recovery kinetics and even suppressed the accelerating effect of bicarbonate, consistent with a direct, modulatory action of bicarbonate on Ca^2+^- dependent, membrane guanylate cyclase activity.

## Introduction

Rod photoreceptors in the retina are highly specialized, unipolar neurons that provide for vision by producing an electrical signal in response to light (reviewed in Hofmann and Lamb, 2022; Kawamura and Tachibanaki, 2022; Lamb, 2022). In darkness, cGMP levels in the outer segment maintain a fraction of the cyclic nucleotide-gated (CNG) cation channels in the open state, allowing Na^+^ and Ca^2+^ to enter. Photoactivated rhodopsin (R*) couples to the G protein transducin to activate PDE, that hydrolyzes cGMP, thereby closing CNG channels. Blockade of the circulating or “dark” current hyperpolarizes the rod and inhibits the vesicular release of the neurotransmitter glutamate.

The phototransduction cascade is regulated by Ca^2+^-dependent feedback. In darkness, Ca^2+^ levels are relatively high. Ca^2+^-bound recoverin sequesters rhodopsin kinase (RK), effectively lowering the fraction available to phosphorylate R* in preparation for full quench of the R* by arrestin binding. Ca^2+^ bound to GCAPs suppresses the activity of membrane guanylate cyclases (ROS-GCs), restraining synthesis of cGMP and limiting the number of CNG channels in the open state. With Ca^2+^ bound, calmodulin decreases the affinity of CNG channel for cGMP, further reducing the number of open CNG channels. In response to light, Ca^2+^ entry is prevented by closure of CNG channels, but its extrusion by a Na^+^/Ca^2+^-K^+^ exchanger continues, bringing about a light-induced fall in intracellular Ca^2+^. Lowered Ca^2+^ accelerates R* phosphorylation, stimulates cGMP synthesis by ROS-GCs, and enhances the affinity of the CNG channel for cGMP. These regulatory mechanisms shape the responses to flashes in darkness and play important roles in light adaptation.

Bicarbonate provides additional cascade modulation; it stimulates ROS-GCs to synthesize cGMP at a faster rate (Mendez et al., 2001; Duda et al., 2015). As a result, the maximum response is larger. Because stimulation of ROS-GCs is greater when Ca^2+^ levels are low, bicarbonate also quickens flash response recovery. Systematic studies of the effects of bicarbonate on rods have been done exclusively on salamander and toads. Here, we wanted to see how bicarbonate impacts the physiology of mammalian rods, which are considerably smaller in size, operate at higher temperature, and generate faster photon responses.

## Materials and methods

*Gnat2^cpfl3/cpfl3^* mice, hereafter referred to as *cpfl3* mice, were purchased from The Jackson Laboratory (Bar Harbor, ME, United States) and used to establish a colony at Boston University. Mice were housed, bred, and handled according to IACUC guidelines. In these mice, a missense mutation in the α-subunit of the cone transducin (*GNAT2*) gene leads to a poor cone-mediated response at 3 weeks that becomes undetectable by 9 months. Rod responses are initially normal but progressively decline with age (Chang et al., 2006; Chen et al., 2020). Mice were kept under a 12 h light/12 h dark cycle with free access to food and water. Both male and female mice were used at ages of 3–7 months. Mice were dark-adapted for 12 h before use. Retinas were isolated under infrared illumination. One retina was used immediately for recording while the other was incubated in Ames’ bicarbonate at 35°C, bubbled with 95% O_2_/5% CO_2_ for up to 90 min prior to recording.

Ames’ medium was prepared from powder obtained commercially (A1420, Sigma Aldrich, St. Louis, MO, United States). Such Ames’ contained either 20 mM bicarbonate or equimolar Cl^−^ in place of bicarbonate. For other types of experiments, 200 μM acetazolamide (Sigma-Aldrich) was added to the Ames’ medium that did not contain bicarbonate. For *ex vivo* electroretinographic recording, an isolated retina was mounted on a perfusion chamber with the photoreceptor side up (Vinberg et al., 2014) and perfused with Ames’ solution at a constant rate of 4 mL/min at 35°C. 50 μM DL-AP4 (Tocris Bioscience, Bristol, United Kingdom) and 100 μM BaCl_2_ (Sigma-Aldrich) were added to block post-photoreceptoral and glial responses, respectively. Solutions were equilibrated with 95% O_2_/5% CO_2_ at pH 7.4. In some experiments, the pH was raised to values between 8.4 and 9.2. Transretinal potentials were recorded with two Ag/AgCl electrodes (WPI, Sarasota, FL, United States), one located on the photoreceptor side of the retina and the other on the ganglion cell side, connected to a differential amplifier (Warner Instruments LLC, Hamden, CT, United States) and an integrated patch amplifier (IPA, Sutter Instruments, Novato, CA, United States). The recordings were low pass filtered at 300 Hz (−3 dB, 8 pole Bessel), and digitized at 5 kHz using Igor Pro (v.9.0.0 64 Bit, Wavemetrics, Portland, OR, United States).

Light from a halogen lamp passing through a 500 nm interference filter (20 nm full bandwidth at half-maximal transmission) was used to stimulate the retina. Intensity was attenuated using neutral density filters. Light intensity was calibrated with a photodiode amplifier (PDA200C, ThorLabs, NJ, United States) and a 200 μm pinhole (Edmund Optics, NJ, United States). Flashes at 500 nm that were nominally 20 ms in duration varied in strength from 0.29 to 127,746 photons μm^−2^.

Figures show averages obtained from as many as six retinas in bicarbonate experiments and from as many as seven retinas in pH experiments, but analyses and statistical tests were carried out on the results of individual retinas. Paired *t*-test for matched pairs or ANOVA followed by a *post hoc* Tukey’s test (Excel 2022) was used to assess whether treatment with bicarbonate and/or higher pH changed the responses. Curve fitting was carried out using Igor Pro.

## Results

**Figure 1:**
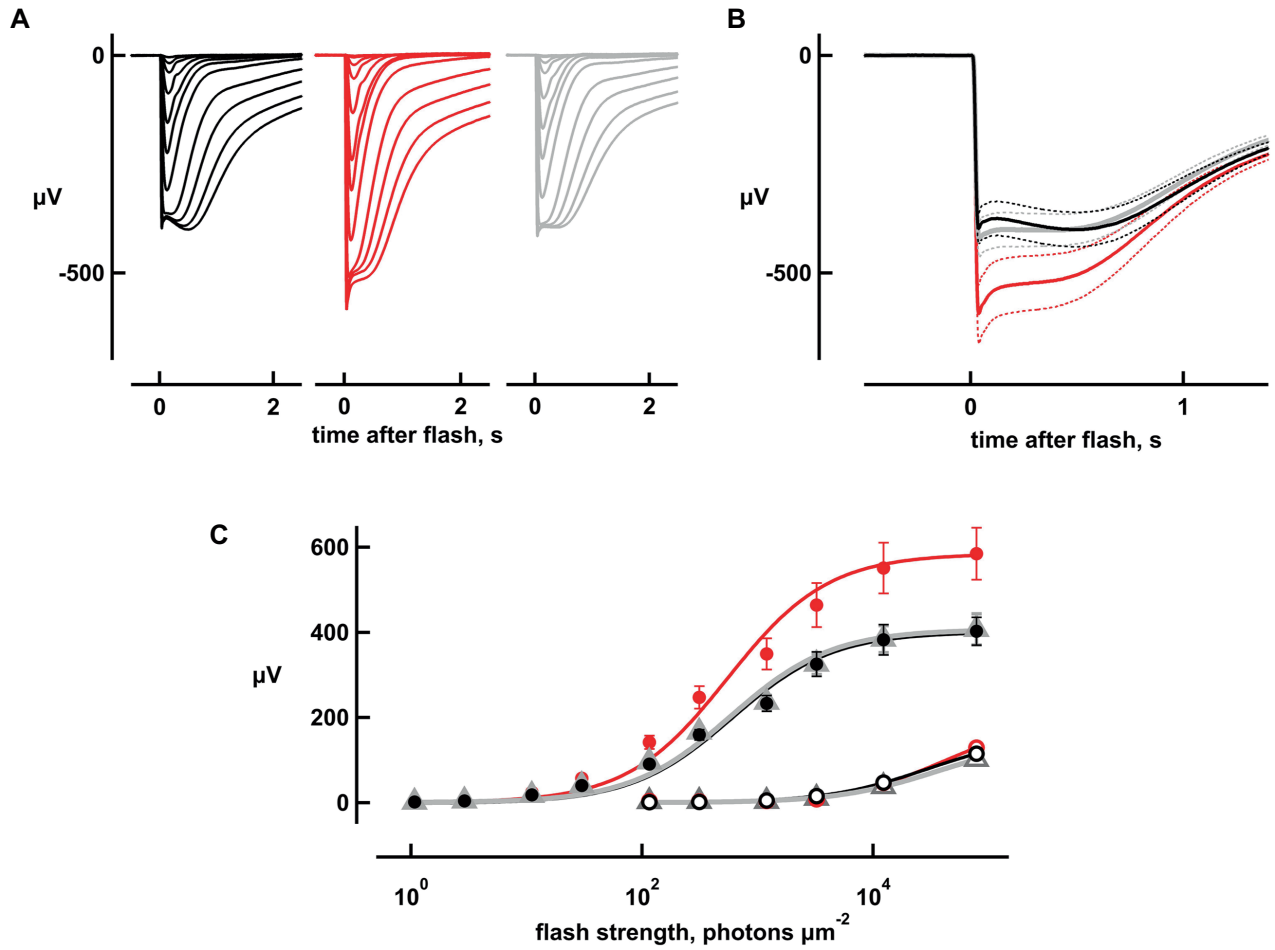
Increases in flash response amplitude and dynamic range with 20  mM bicarbonate. Traces are averaged responses of rods from *cpfl3* mouse retinas exposed to flashes of increasing strength during pre-treatment with Ames’ medium that did not contain any added bicarbonate (black traces), during exposure to bicarbonate (red traces), and during wash with the Ames’ medium lacking bicarbonate (gray traces). **(A)** Flash response families. Traces are averaged responses of six retinas for which there were up to five trials per flash. Flash strengths ranged from 1 to 127,746 photons μm^−2^ at 500  nm. **(B)** Averaged responses to the brightest flash, redrawn from **(A)**. Maximum response amplitude increased from 400 to 600 μV with bicarbonate and subsided to 420 μV upon washout. Dotted lines delineate ± SEM. **(C)** Stimulus–response relation. Response amplitudes from **(A)** were plotted against flash strength for perfusions with (red) or without bicarbonate (black, gray) at their peaks (filled symbols) and at 2 s after the flash (open symbols). For the latter, values for the dimmer flashes were omitted for clarity. Error bars plot SEM, which was exceedingly small for open symbols. Results were fit with the Michaelis–Menten function: *r* = *r*_max_*i*/(*i* + *i*_1/2_) where *i*_1/2_ is the flash strength eliciting a half-maximal response.

Figure 1 shows averaged responses from isolated, *cpfl3* mouse retinas to a series of flashes of increasing strength from electroretinogram (ERG) recordings. The ERG is a massed field potential that summates the electrical responses of retinal neurons to light. Here, the responses were generated almost entirely by rods; cones in this retina were several log units less sensitive than normal (Chen et al., 2020) and at the highest flash strengths, were unlikely to have contributed more than 6% to the flash responses. Glial responses and synaptic transmission were blocked pharmacologically (Coleman et al., 1987; Green and Kapousta-Bruneau, 2007). Bicarbonate produced an intensity-dependent increase in flash response amplitude (Figure 1A) that was reversed upon washing with Ames’ medium not containing bicarbonate. During the wash, maximal responses were sometimes smaller than during pre-treatment due to rundown. Results from experiments in which the maximal response during the wash differed from the pre-treatment value by more than 20% were not included.

The absence of added bicarbonate to the perfusate did not necessarily mean that bicarbonate was absent, because at least some types of cones (Musser and Rosen, 1973) and Muller glia (Ochrietor et al., 2005) express carbonic anhydrase in the mammalian retina. Rods do not, but they take up bicarbonate at their synapses (Makino et al., 2019) *via* transport systems (Bok et al., 2003; Kao et al., 2011), by passage through Cl^−^ channels (Qu and Hartzell, 2000) and possibly through gap junctions. To assess whether rod photoresponses were modulated by bicarbonate from endogenous sources under our experimental conditions, retinas were perfused with 200 μM acetazolamide, a carbonic anhydrase inhibitor. No differences in flash response amplitude or kinetics were observed (*n* = 4, Supplementary Figure S1), confirming the lack of carbonic anhydrase activity in mouse rods and ruling out any significant basal uptake of endogenous bicarbonate.

The maximal, saturating rod response to a bright flash (*r*_max_) provided an indirect measurement for the magnitude of the dark current, which was proportional to the number of CNG channels in the open state. However, there was a “nose” in the response that arose from changes in the activities of voltage-dependent ion channels in the inner segment (Bader et al., 1982; Vinberg et al., 2009). The nose was more prominent with bicarbonate (Figures 1A,B), because the larger dark current maintained the resting membrane potential at a more depolarized level in darkness and supported a larger current through voltage-gated K^+^ channels. The delayed closure of a larger population of these channels during the flash response then caused a greater reduction in K^+^ efflux at the peak of the response, causing a larger drop in amplitude to a plateau. Closure of these voltage-gated K^+^ channels, as well as opening of cation channels activated by hyperpolarization meant that it was only possible to estimate a lower limit for the dark current in each retina. Maximum response amplitudes were increased by 46 ± 5% (mean ± sem, *n* = 5, *p* = 0.0044; Figures 1A,B), with 20 mM bicarbonate.

Although *r*_max_ was larger with bicarbonate, rod saturation occurred at approximately the same flash strength (Figure 1C). The *i*_1/2_ value, which is the flash strength giving rise to a half-maximal response and is inversely proportional to relative sensitivity, decreased slightly, by 3.6 ± 1% (*n* = 6, *p* = 0.0174) with bicarbonate. The slowly rising “tail,” that was especially notable in the recovery phase of the responses to the brighter flashes, appeared to be largely unchanged by bicarbonate (Figures 1A,B). The tail summates aberrant single photon responses that are thought to arise from the rare failure of some photoactivated rhodopsins to be phosphorylated and shut off properly (Baylor et al., 1984; Chen et al., 1995, 1999; Kraft and Schnapf, 1998). Preservation of the dependence of tail amplitude on flash strength upon treatment with bicarbonate (Figure 1C) may suggest that bicarbonate had little or no influence over the incidence, amplitude, and duration of aberrant responses.

**Figure 2:**
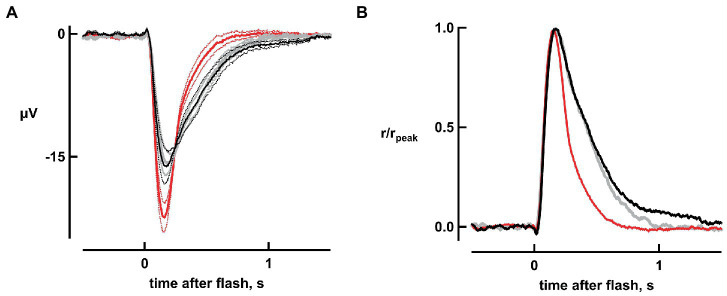
Improved absolute sensitivity and faster flash response recovery with bicarbonate. **(A)** Averaged responses to a flash of 11 photons μm^−2^, replotted from Figure 1A. Black and gray traces represent pre-treatment and wash respectively, while the red trace represents the response during exposure to bicarbonate. Dotted lines delineate ± SEM. **(B)** Acceleration of response recovery with bicarbonate. Averaged responses to the four dimmest flash strengths were divided by their peak amplitudes and then averaged for each retina. Averages were then taken for six retinas to compare the response kinetics in the presence of bicarbonate (red trace) to those during pretreatment (black trace) and those taken during washout of bicarbonate (gray trace).

The four dimmest flash response amplitudes (which were less than 15% of maximum, and hence were within the linear range) increased by 44, 28, 38, and 43% with bicarbonate. Figure 2A depicts the results for one flash strength. Overall, absolute sensitivity, assessed by the responses to dim flashes, increased with bicarbonate by 38 ± 7% (*n* = 6, *p* = 0.0018; Figure 2A). Therefore, dynamic range was extended to dimmer flashes by bicarbonate.

As is evident in Figure 2, bicarbonate quickened flash response kinetics. To quantify changes in the single photon response, we measured integration time, given by the response integral (area under the curve) of the response normalized to its peak, for dim flash responses that were less than 15% of the maximal. Integration time shortened by 65 ± 2% (*n* = 6, *p* = 0.000005) in bicarbonate (Figure 2B) without any change in time to peak.

**Figure 3:**
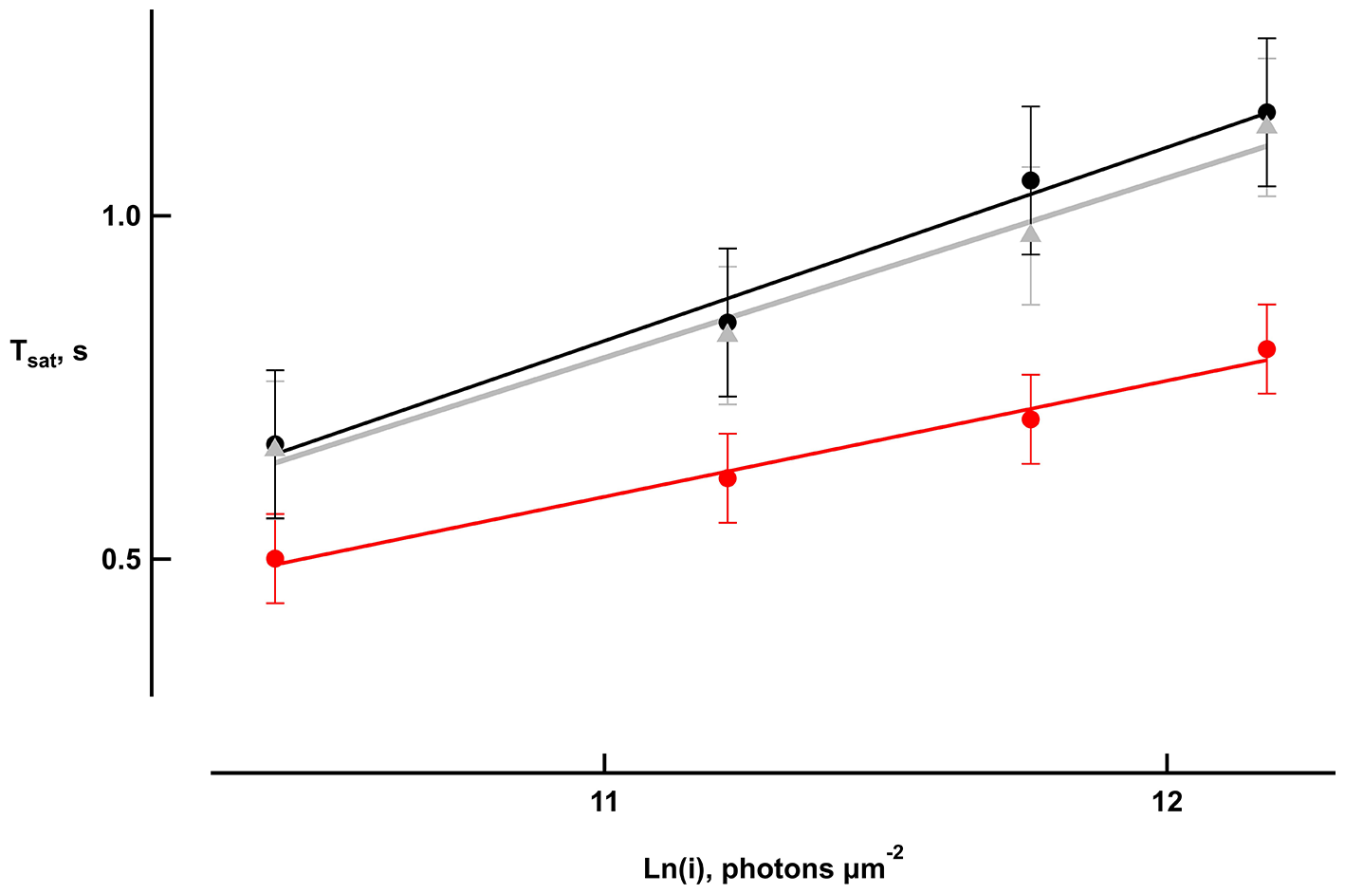
Reduced saturation time of bright flash responses with bicarbonate. Saturation time, T_sat_, was measured from mid-flash to 25% recovery of the response peak in six retinas. Slopes of the saturation functions from linear regressions were: 282 ms pretreatment (black), 169 ms with bicarbonate (red), and 261 ms after washing (gray).

Bicarbonate also shortened the time that bright flash responses remained in saturation, as shown in “Pepperberg plots” that graph response saturation time (*T*_sat_) as a function of the natural logarithm of flash strength (Figure 3). Linear regressions yielded an average slope of 271 ± 21 ms (*n* = 6) during pretreatment that dropped to 165 ± 11 ms upon exposure to bicarbonate. The slope recovered to 250 ± 24 ms after washing for a duration exceeding 30 min. The change in slope induced by bicarbonate, when compared to the averages of pretreatment and wash for each retina, was significant (*p* = 0.0006).

**Figure 4:**
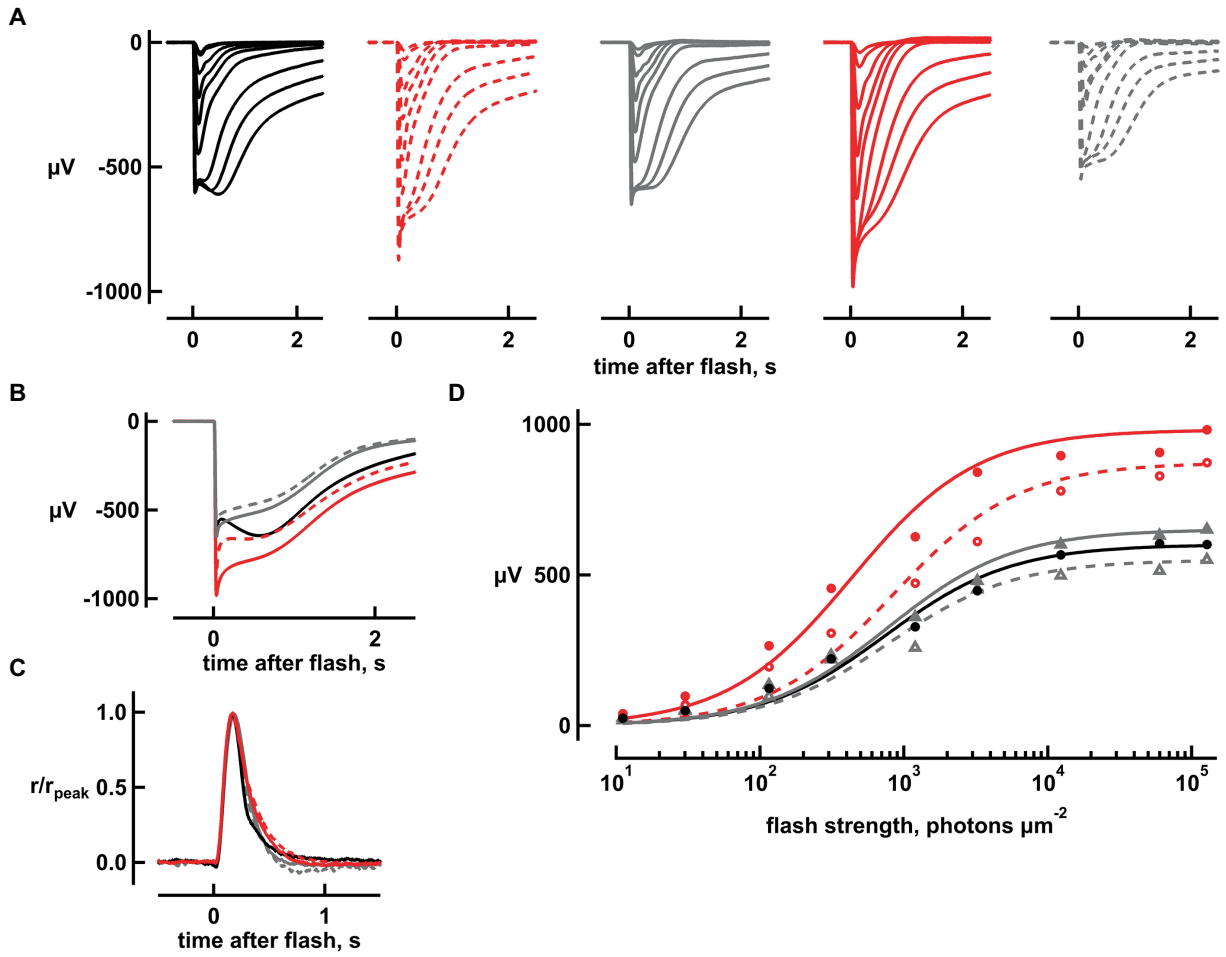
Increases in flash response amplitude and dynamic range but little change in flash response kinetics, upon raising pH to 8.4. **(A)** Flash response families. Traces are averaged responses of rods from seven retinas for which there were up to five trials per flash during pretreatment with Ames’ medium at pH 7.4 that did not contain any added bicarbonate (black traces), during exposure to high pH without bicarbonate (red dashed traces), during first wash (gray traces), during exposure to 20 mM bicarbonate at high pH (red continuous traces), and during second wash (gray dashed traces). Flash strengths ranged from 1 to 127,746 photons μm^−2^ at 500 nm. **(B)** Averaged responses to the brightest flash, redrawn from **(A)**. Maximum response amplitude increased from 650 to 850 μV with high pH and returned to 640 μV upon washout. In the presence of bicarbonate and high pH, amplitude increased to 900 μV which subsided to 510 μV upon final washout. **(C)** Little change in dim flash response kinetics with high pH or with bicarbonate plus high pH. Averaged responses to the two dimmest flash strengths were divided by their peak amplitudes and then averaged for each retina. Averages were then taken for five retinas. **(D)** Stimulus–response relation. Response amplitudes from **(A)** were plotted against flash strength for perfusions at pH 7.4 without bicarbonate (black, gray filled, gray open symbols), at high pH (red open symbols) and at high pH with 20 mM bicarbonate (red filled symbols). Results were fit with the Michaelis–Menten function.

To understand if the changes brought by bicarbonate were due to it causing a rise in intracellular pH or to a direct influence on the phototransduction cascade, retinas were perfused with Ames’ at pH values ranging from 8.4 to 9.2. At pH 8.4, flash response amplitudes were increased (Figures 4A,B); the response to the dimmest flash increased by 31 ± 5% (*n* = 6, ANOVA *p* = 0.006057) and the maximal, saturating response increased by 37 ± 5% (*n* = 7, *p* = 0.00048) with an enlarged nose (Figure 4B). Response saturation was reached at a similar flash strength and the *i*_1/2_ at pH 7.4 did not change at pH 8.4 (Figure 4D). All of these effects were similar to those produced by 20 mM bicarbonate, but a marked difference was that raising pH to 8.4 did not reduce integration time of the dim flash response nor did it reduce *i*_1/2._ One retina was subjected to a series of pH elevations from 7.4 to 8.4, 8.8, 9.2, 8.8, and 8.4, before returning to 7.4. The pH was raised from 7.4 to 9.0 in two other retinas. In general, the maximal response was greater than normal at all elevated pH settings (*p* = 0.018, *n* = 10), as was integration time for the dim flash response (*p* = 0.0001; Supplementary Figure 2). The magnitude of the change did not appear to vary systematically over the pH range 8.4 to 9.2 for either parameter.

Application of 20 mM bicarbonate accentuated the effects of pH elevation. When bicarbonate was applied at pH 8.4, the increases in maximal response of 64 ± 6% (*n* = 6, *p* = 0.000003) and sensitivity to flashes of 76 ± 10% (*n* = 6, *p* = 0.0008619) were significant and were greater than those produced by raising pH alone for absolute sensitivity (*p* = 0.000051) and maximum response (*p* = 0.007609). Moreover, bicarbonate at pH 8.4 slowed dim flash response recovery by 16 ± 5% (*n* = 6; *p* = 0.01317), and lowered i_1/2,_ increasing the relative sensitivity on average by 5.6 ± 1% (*n* = 6, *p* = 0.02335; Figure 4D). The effects of bicarbonate at pH 9.0 also appeared to be greater than high pH alone in two retinas. The effect of bicarbonate at high pH on saturation time of responses to bright flashes was the same as that at high pH alone.

## Discussion

Amphibian rods readily take up bicarbonate at their synapse, whereupon it diffuses throughout the cytosol, finally exiting from the outer segment by the action of a bicarbonate/chloride exchange (Koskelainen et al., 1994; Makino et al., 2019). Within the rod, bicarbonate opens a greater fraction of CNG channels and increases the dark current. In ERG recordings of amphibian rods, 6 mM bicarbonate replacing either phosphate or HEPES increased the dark current by 1.35–1.4-fold (Donner et al., 1990). In that study, there was no effect in single cell recordings of salamander rods, likely because the synapse was lost during tissue preparation, precluding bicarbonate uptake. In subsequent single cell recordings of salamander rods with an intact synapse, there were increases in dark current by 1.2-fold with 30 mM bicarbonate and by 1.6-fold with 50 mM bicarbonate (Duda et al., 2015). These studies substituted bicarbonate for phosphate. Smaller, 1.1–1.2-fold increases in dark current were observed for single rods when 25–50 mM bicarbonate was substituted for MOPS (Duda et al., 2015; Makino et al., 2019). In the present study of mouse rods, bicarbonate was substituted for equimolar Cl^−^. The maximal rod response as measured by the electroretinogram, which is proportional to the dark current, increased by more than 1.4-fold with bicarbonate (Figure 1). Variability in the efficacy of bicarbonate in enhancing dark current is therefore affected by the composition of the perfusion medium and perhaps, by species differences.

Lamb et al. (1981) found that responses to dim flashes in toad rods were diminished by 22 mM bicarbonate, but their bicarbonate solution also raised extracellular Ca^2+^ concentration by two-fold, which would have suppressed ROS-GC activity. With extracellular Ca^2+^ levels held constant, dim flash responses were approximately 2-fold larger for frog rods when 6 mM bicarbonate replaced phosphate (Donner et al., 1990), but there was no change for salamander rods when 30 mM bicarbonate replaced MOPS (Duda et al., 2015) or Cl^−^ (Makino et al., 2019). Flash sensitivity was approximately 1.4-fold higher in monkey rods when 20 mM bicarbonate replaced Cl^−^, based on a comparison of 18 rods in bicarbonate to four other rods in the absence of bicarbonate (Baylor et al., 1984). In the present study of mouse rods, the response to dim flashes also increased 1.4-fold with bicarbonate (Figure 2). Enlargement of the dim flash response by bicarbonate improved flash sensitivity, and in doing so, it expanded the dynamic range of rod responsivity to dimmer flashes. In support of a role for bicarbonate in rods of the intact eye in the living animal, knockout of carbonic anhydrase XIV in mice led to a 0.4-fold decline in bright flash ERG a-wave amplitude and a 0.3-fold decline in b-wave amplitude. Lacking any significant changes in the number of rods in the retina or in rod outer segment length (Ogilvie et al., 2007), the diminished ERG was presumably due to a reduction in dark current in the rods.

In primate and amphibian rods, time to peak is faster and integration time is shorter for dim flash responses when bicarbonate is present (Baylor et al., 1984; Duda et al., 2015), but only the latter was true for mouse rods in the present study. The briefer integration time would improve temporal resolution but would diminish sensitivity to steady illumination.

Bicarbonate binds the core catalytic domain of ROS-GCs to stimulate cGMP synthesis at a faster rate. Stimulation is even more powerful at low Ca^2+^ levels (Duda et al., 2015; Makino et al., 2019). The ensuing rise in cGMP concentration supports the opening of a greater fraction of CNG channels to increase the dark current. Alkalinization of amphibian rods (Kalamkarov et al., 1996), due to bicarbonate combining with a proton to eventually form CO_2_, also enhances the dark current (Liebman et al., 1984; Duda et al., 2015). To explore the mechanism of action of bicarbonate on the flash response in murine rods, retinas were perfused with Ames’ medium at high pH, to raise intracellular pH (Saarikoski et al., 1997), with and without bicarbonate. Elevating pH above 8.4 reproduced the effects of 20 mM bicarbonate at pH 7.4 by increasing dark current and sensitivity to flashes, but differed from bicarbonate in that it failed to accelerate dim flash response kinetics (Figures 1, 4; Supplementary Figure S2). Raising extracellular pH to 8.4 even caused bicarbonate to slow dim flash recovery. ROS-GC activity is not pH dependent over the range 7.4–9 (Fleischman and Denisevich, 1979; Duda et al., 2015), therefore, direct stimulation of ROS-GC activity by bicarbonate is critical for its physiological effects on the flash response.

Increasing flash strength beyond that required for a maximum response caused responses to remain in saturation progressively longer before recovering. Bicarbonate shortened saturation time (Figure 3), because it accelerated cGMP synthesis and exerted a pH effect that supported CNG channel opening (Liebman et al., 1984; Duda et al., 2015). In Pepperberg plots, the slope of the relation, τ_D_, estimates the time constant of the rate-limiting step in the flash response recovery (Pepperberg et al., 1992), namely the hydrolysis of GTP by transducin bound to PDE (Krispel et al., 2006). Interestingly, the τ_D_ of ~270 ms in the absence of bicarbonate shortened to 160 ms with bicarbonate. A value of ~200 ms was reported previously for mouse rods perfused with bicarbonate by many other groups (e.g., Calvert et al., 2000; Chen et al., 1999) although shorter values have sometimes been observed (e.g., Chen et al., 2010, 2012; Woodruff et al., 2014). We attribute the slower τ_D_ in the present study and the apparent slowing of τ_D_ in the absence of bicarbonate to the intrusion of aberrant single photon responses. Aberrant responses produced the tail, slowed the time course of the early recovery phase, and extended the time in saturation of the bright flash response (Figures 1A, B). Since their number and hence the overall magnitude of their effects increased with flash strength, aberrant responses inflated the slope of the Pepperberg relation. Constancy of aberrant response size (Figure 1B) meant that there was a relatively weaker prolongation of the *T*_sat_ of the responses enlarged by bicarbonate, when measured at a criterion level of response recovery. Another possibility was that bicarbonate raised pH (Kalamkarov et al., 1996) to a level that slowed the rate of Ca^2+^ extrusion (Hodgkin and Nunn, 1987; Schnetkamp, 1995) and delayed Ca^2+^ feedback onto ROS-GC activity. The extension of saturation time would have had a greater relative impact on the responses at the lower end of the flash strength range. Other mechanisms that cannot yet be ruled out include an acceleration of GTP hydrolysis by transducin as a direct effect of bicarbonate or by the bicarbonate-induced alkalinization.

## Data availability statement

The raw data supporting the conclusions of this article will be made available by the authors, without undue reservation.

## Ethics statement

The animal study was reviewed and approved by Boston University's Institutional Animal Care and Use Committee (IACUC).

## Author contributions

RA and CM designed the study. RA carried out the experiments, collected data, and analyzed results. AK participated in some experiments and helped with analysis. RA wrote the first draft of the report. RA, CM, and MC edited and approved the final report. All authors contributed to the article and approved the submitted version.

## Funding

This work was supported by National Eye Institute (EY01157, EY031702).

## Conflict of interest

The authors declare that the research was conducted in the absence of any commercial or financial relationships that could be construed as a potential conflict of interest.

## Publisher’s note

All claims expressed in this article are solely those of the authors and do not necessarily represent those of their affiliated organizations, or those of the publisher, the editors and the reviewers. Any product that may be evaluated in this article, or claim that may be made by its manufacturer, is not guaranteed or endorsed by the publisher.
