# Supplementary material for: Bicarbonate boosts flash response amplitude to augment absolute sensitivity and extend dynamic range in murine retinal rods
Source: Front Mol Neurosci. 2023 Apr 14;16:1125006. doi: 10.3389/fnmol.2023.1125006 (PMC10140344; doi:10.3389/fnmol.2023.1125006)
Supplement: Supplementary file 1 [file Image_1.pdf]

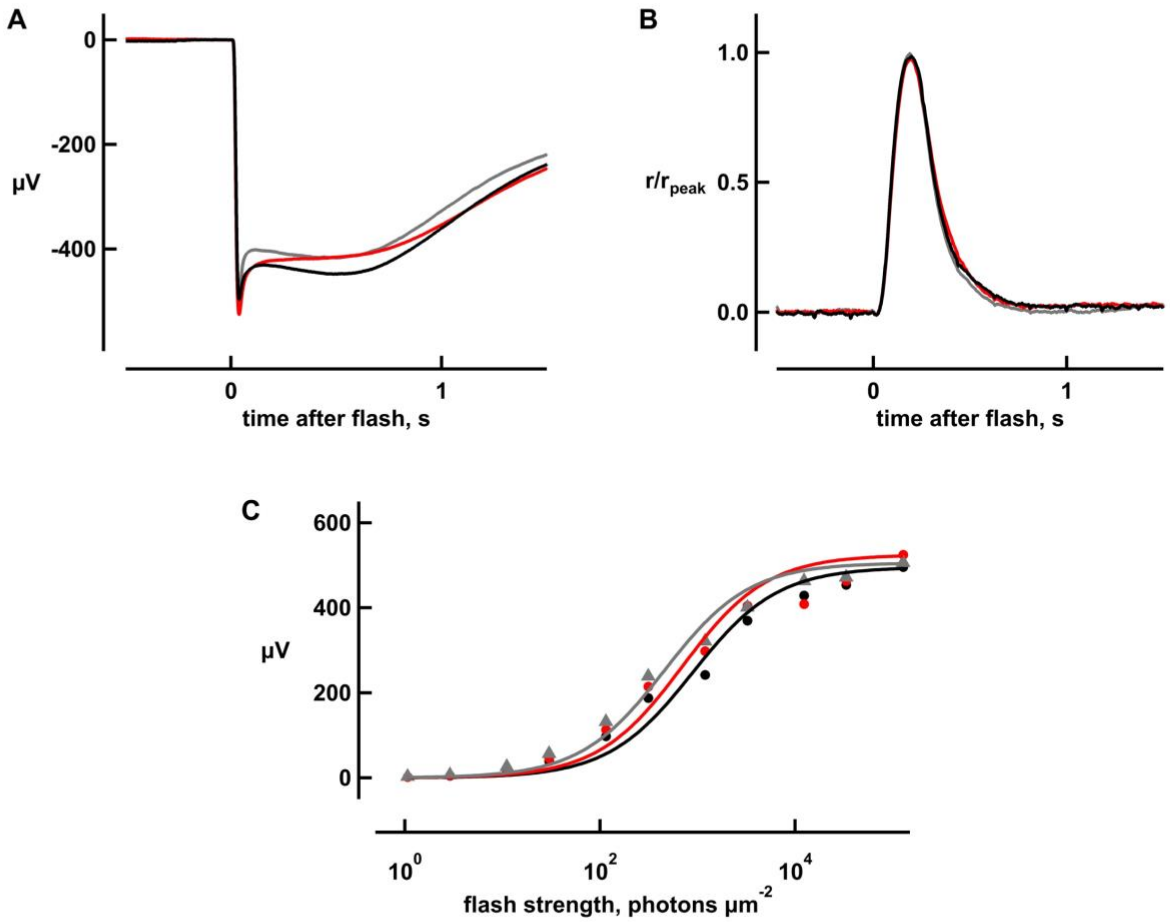

Supplemental Figure S1. No effect of a carbonic anhydrase inhibitor on rod responses to flashes. Traces are averaged responses of rods from four *cpfl3* mouse retinas exposed to flashes during pre-treatment with Ames' medium that did not contain any acetazolamide or added bicarbonate (black traces), during exposure to 200 mM acetazolamide (red traces), and during wash (gray traces). (A) No significant change in the response to a saturating flash. (B) No change in dim flash response kinetics with acetazolamide. Averaged responses to the four dimmest flash strengths were divided by their peak amplitudes and then averaged for four retinas. (C) No changes in the sensitivity to flashes with acetazolamide. Stimulus-response relations were fit with the Michaelis-Menten function.
