# Supplementary material for: Bicarbonate boosts flash response amplitude to augment absolute sensitivity and extend dynamic range in murine retinal rods
Source: Front Mol Neurosci. 2023 Apr 14;16:1125006. doi: 10.3389/fnmol.2023.1125006 (PMC10140344; doi:10.3389/fnmol.2023.1125006)
Supplement: Supplementary file 2 [file Image_2.pdf]

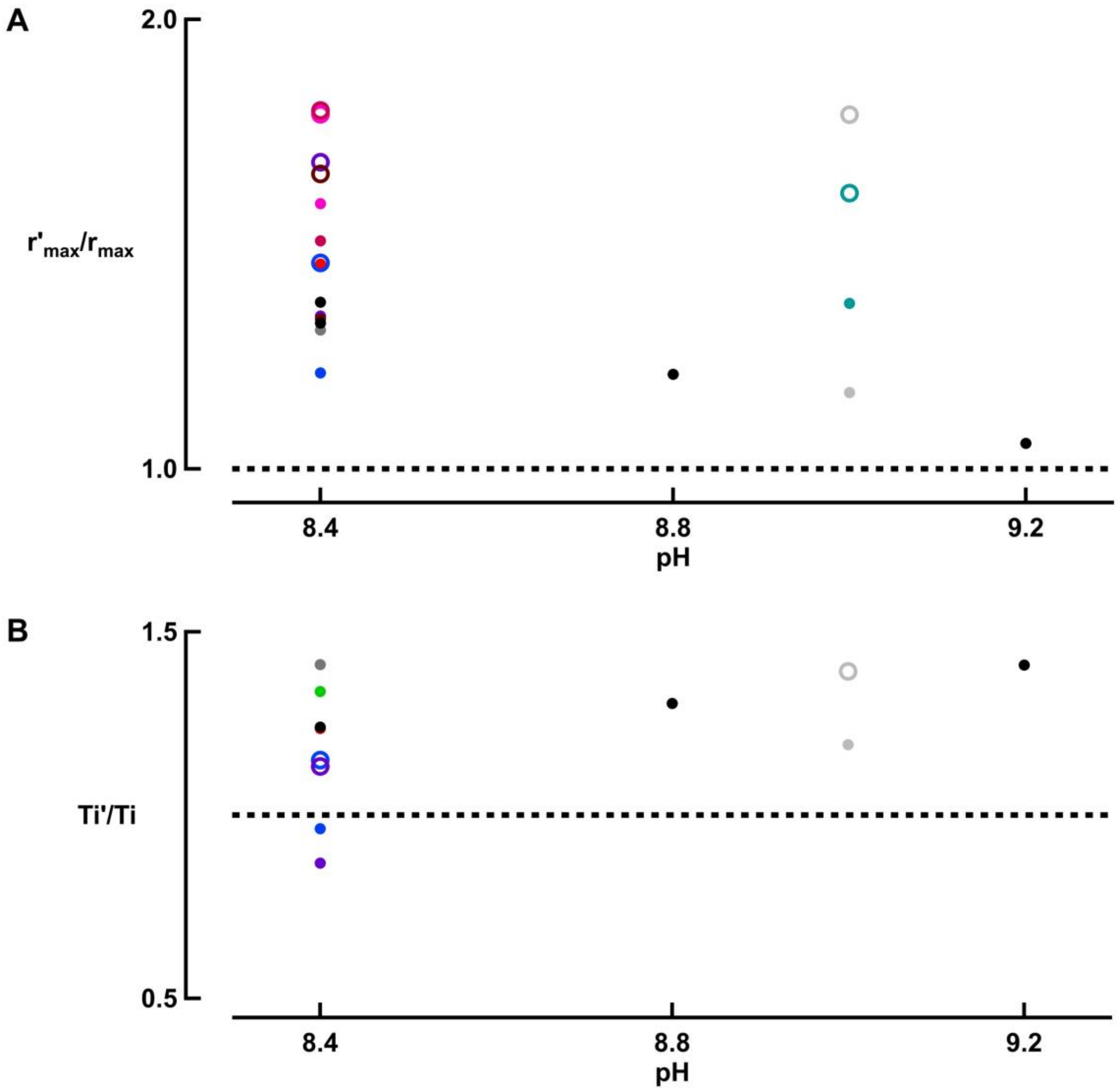


Supplemental Figure S2. Increased maximal response amplitude and slower dim flash response recovery at high pH. Dashed horizontal lines at 1.0 show the ratio expected for the absence of a pH effect. Results obtained from one retina after multiple pH changes are shown in black. Individual retinas are represented by different colors. Open symbols represent responses during treatment with 20 mM bicarbonate for several of the same retinas. (A) Maximal response at high pH ($r'_{max}$) relative to that at pH 7.4 ($r_{max}$), was pH invariant. (B) Prolongation of integration time did not differ over the pH range 8.4 to 9.2. Integration times were normalized by taking the ratio at high pH (Ti') divided by the integration time at pH 7.4 (Ti).
